# Therapeutic Notes

**Published:** 1896-08-01

**Authors:** 


### THERAPEUTIC NOTES.

### HOT AIR AS A THERAPEUTIC AGENT.

In January of this year there appeared among our book notices a review of a pamphlet dealing with the "Tallerman-Sheffield method of localised hot air treatment." Since that date the experiences of other observers have been placed on record, and the success which has attended the application of this new method warrants more detailed notice. Speaking generally, the method depends on the exposure of a limb or limited portion of the body to dry air heated to a high temperature; in fact, on their exposure to the atmosphere of a Turkish bath. The apparatus, which is different in form and shape according as the part under treatment differs in external configuration, has more or less the character of a copper cylinder; it is, however, lined with asbestos, and has other means of protecting the skin from direct heat by the copper, and there are arrangements for adjusting the air supply The cylinder is heated by gas jets arranged underneath. The arm, leg, or other portion of the body is placed in the chamber, and the temperature gradually raised from about 160 to 280 degrees. Owing to the dryness of the air a temperature of this height can generally be tolerated without any discomfort. The operation is generally continued for about forty minutes, and may be repeated every day or oftener. The class of cases which appear to lend themselves most happily to this treatment are acute gout, rheumatoid arthritis, chronic rheumatism, pyæmic rheumatism, sprains and flat-foot, while in the treatment of old adhesions, subsequent to their being broken down, the method appears to be attended with great relief of pain. The therapeutic effect of this method may be considered under two headings—(1) local; (2) general. With regard to the first, the results observed are increased blood flow to the part with redness, copious diaphoresis, relaxation of the tissues with increased range of movement and relief of pain. With regard to the general results, the temperature rises some two degrees, the pulse becomes fuller, and slightly more frequent. There is general diaphoresis and relief of pain.

The benefits resulting from this treatment are largely due to the vascular dilatation and improved circulation, not only in the part immediately exposed to the high temperature, but generally throughout the system. Counter-irritation and reflex influences may take some share in the matter. The rise in temperature is an interesting phenomenon, and at first appears in contradiction to physiological teaching. Although not explained by any of the observers who have used this method, it is probably due to imperfectly co-ordinated diaphoresis in parts of the body other than that exposed to the direct heat of the bath, that is to say the blood as it flows through the heated area is not completely cooled down before it passes on to other areas, where the conditions are different and where reflex superficial vascular dilatation and consequent diaphoresis are not correspondingly established. The improvement in condition is by no means confined to the part locally treated; for instance, in rheumatoid arthritis the pain is alleviated, and the range of movement increased in distal joints also, and this not only during but after the treatment. Many of the published cases show very remarkable results, and this is especially so in cases of acute gout and rheumatoid arthritis. Appended will be found references to papers and published cases dealing with this subject:—

[1] Alfred Willett, F.R.C.S., Clinical Journal, May 31, 1894. [2] J. E. Sergeant, Lancet, Jan. 12, 1895. [3] K. Sibley. Lancet. June 6, 1896. [4] W. Walsham, F.R.C.S., "Deformities of the Human Foot with their Treatment." [5] "The Tallerman-Sheffield Patent Localised Hot Air Bath." A pamphlet printed by Baillière, Tindal, and Cox.

### FORMALIN.

Formalin, or formol, which is an aqueous solution of formic aldehyde, must undoubtedly be regarded as one of the most powerful of known antiseptics, but owing to its necrotic action on the skin it has not been found suitable for most surgical purposes or for wound treatment. It has, indeed, been recommended as a spray in diphtheria when in 2 per cent. solution; but according to K. Walter, it might be used in a far greater degree of dilution for this purpose, since experimentally it has been known to kill the diphtheria bacillus in a strength of 1 in 10,000. The undoubted advantages that formalin possesses, apart from its necrotic action, has simultaneously led to a series of experiments by independent observers, in the hope that some of the difficulties associated with its use might be overcome. K. Walter, experimenting in the research department of the 10th German Army Corps, has conducted a series of observations as to the strength at which formalin is fatal to the common forms of pathogenic bacteria. He has found that for anthrax, cholera, typhoid, staphylococcus, and diphtheria a strength of 1 in 10,000 is sufficient to arrest growth, and in slightly stronger solutions suffice to destroy. For the rapid sterilisation of solid materials it is sufficient to moisten the outer surface and place in an air-tight chamber for a few hours. The vapour pervades the material and renders it sterile. Leather goods can be thoroughly disinfected in twenty-four hours without being damaged. Fæces can be deodorised by a 10 per cent. solution, or rendered aseptic by a 10 per cent. solution in ten minutes. Hence, for field operations, and general disinfecting purposes in war, formalin has much to recommend it both from the smallness of its bulk and from the rapidity of its action. For surgical purposes, two discoveries have recently been made which should clear the way for its more universal application as an antiseptic. The first has been described by Schleick,[2] and is a means of applying formalin to wounds. His experiments, it is true, were not carried out on the human subject, but in the case of animals his experience is extensive. The method is to combine formalin with gelatin, and then prepare it in the form of a powder. In this way, when dusted on recent wounds or cuts, it appears to encourage healing by first intention, and to prevent all septic processes. We shall look forward to the results when this new preparation is applied to the case of human beings. Holbau and Hlawacek[3] have made the second of these useful discoveries, for they have described how catgut can be safely sterilised with formalin and finally freed from any excess of this antiseptic. The catgut must be soaked in 5—10 per cent. solution of formalin for twelve hours, then boiled in water half an hour, and finally dipped in alcoholic solution of corrosive sublimate.

[1] Zeitsch für Hyg. und Infecktionskr, vol. 21, p. 421. [2] Therapeut. Monatscrif, Feb., 1896. [3] Therapeutic Monatschrift, March, 1896.

